# Autophagy-mediated regulation patterns contribute to the alterations of the immune microenvironment in periodontitis

**DOI:** 10.18632/aging.202165

**Published:** 2020-12-03

**Authors:** Xiaoqi Zhang, Yu Jin, Qingxuan Wang, Fan Jian, Minqi Li, Hu Long, Wenli Lai

**Affiliations:** 1 Department of Orthodontics, West China Hospital of Stomatology, State Key Laboratory of Oral Diseases, Sichuan University, Chengdu 610041, China; 2 Department of Bone Metabolism, School of Stomatology, Shandong University and Shandong Key Laboratory of Oral Tissue Regeneration and Shandong Engineering Laboratory for Dental Materials and Oral Tissue Regeneration, Jinan 250014, China

**Keywords:** autophagy, immune microenvironment, periodontitis, immunity, gene expression

## Abstract

The relationship between autophagy and immunity has been thoroughly investigated. However, little is known about the role of autophagy in shaping the immune-microenvironment of periodontitis. Thus, we aim to explore the impact of autophagy on the immune-microenvironment of periodontitis. The expression distinctions of autophagy genes between healthy and periodontitis samples have been investigated. The connections between autophagy and immune characteristics including infiltrating immunocyte, immune reaction and human leukocyte antigen (HLA) gene were evaluated. The distinct autophagy-mediated expression patterns were identified and immune characteristics under distinct patterns were revealed. Autophagy phenotype-related genes were identified. 16 autophagy genes were dysregulated and a ten-autophagy classifier was constructed that can well distinguish periodontitis and healthy samples. Immune characteristics were closely related to autophagy: higher expression of EDEM1 positively relates to infiltrating activated B cell; NCKAP1 negatively relates to monocyte; CXCR4 enhances BCR Signaling Pathway and PEX3 decreases the activity of TNF Family Members Receptors; positive expression correlation of EDEM1-HLADOB and negative correlation of RAB11A-HLADOB were observed. Two distinct autophagy expression patterns were identified which demonstrated different immune characteristics. 4309 autophagy phenotype-related genes were identified, and 219 of them were related to immunity, whose biological functions were found to be involved in immunocyte regulations. Our study revealed the strong impact of autophagy on the immune-microenvironment of periodontitis and brought new insights into the understanding of the pathogenesis of periodontitis.

## INTRODUCTION

Periodontitis is a chronic inflammatory disease in periodontium caused by infection from dental biofilm [1]. If not treated properly, periodontitis could cause the formation of the periodontal pocket, absorption of alveolar bone, attachment loss and even tooth loss. Moreover, it has been linked to some systemic diseases such as diabetes, cardiovascular diseases and rheumatoid arthritis [2]. Being the sixth most prevalent health condition, periodontitis has been a great burden for the economy and society due to its high costs of treatment and productivity loss such as pain and chewing problems [3]. It is now widely believed that instead of being a disease resulting from an individual pathogen, periodontitis is the consequence of dysbiosis and the disturbance in the local immune homeostasis [4]. Dysregulation of innate and adaptive immune reactions plays a pivotal role in the etiology of periodontitis. The presence of bacterial antigens initiates the immune and inflammatory responses to eliminate pathogens. However, the long-standing inflammatory responses, especially in older individuals, might trigger T- or B- cell dysregulation, DNA damage, cellular aging, and oxidative stress, potentially immunodeficiency and opportunistic infections [5]. This in turn could provide an opportunity for the invasion of herpesviruses, which results in cytotoxic effects and immunopathology. To suppress this destructive reaction, regulatory T cells and noncoding RNAs seek to retain the recessive inflammation [6].

Recent studies have suggested that autophagy might involve in the pathogenesis of periodontitis, including mediation of the infection in the periodontium, immune and inflammatory responses from the host, as well as alveolar bone metabolism [7]. Autophagy is an evolutionarily highly conserved degradation process through lysosome, during which the cytoplasmic denatured protein, organelle and pathogens are transported into the lysosome for further degradation. There are in general four classes of autophagy, including microautophagy, macroautophagy, chaperone-mediated autophagy, and non-canonical autophagy, the most common form of which is macroautophagy [8]. The process of autophagy starts with the intermediate organelle called the autophagosome, which is a bilayer vesicle that sequestered a small portion of cytoplasm, containing soluble substances and organelles. The autophagosome then fuses with the lysosome to form an autolysosome so that materials can be degraded inside of it [9]. Autophagy is a fundamental pathway for immunity, it is considered to be the primary form of innate immunity for eukaryotic cells against microorganisms. There are four main aspects autophagy plays in immunity: the direct elimination of microorganisms, the regulation of inflammation, the regulation of innate immunity, the regulation of adaptive immunity [10]. Over the years, the fundamental role autophagy has in periodontitis has caught researchers’ attention. As the sensor of infection in cells, dysfunction of autophagy will sabotage the defense of cells against pathogens. Most of the infections would enhance autophagy thus accelerating the elimination of pathogens. However, some of the periodontal pathogens could escape from the identification of autophagy molecules and even interfere with the formation of the autophagosome. For instance, Bélanger M et al. discovered that P. gingivalis could activate autophagy to provide autophagosome for it to hide inside to escape the identification from the host [11]. On the other hand, autophagy as well as innate immunity are regarded as the first line of defense against periodontal pathogens. Under the stimulation of periodontal pathogens, pattern-recognition receptors such as Toll-like receptors and NOD-like receptors recognize pathogen-associated molecular patterns and damage associated molecular patterns, which triggers immune reactions and autophagy to wipe out the microorganisms, while autophagy in turn could regulate the extent of inflammation [12].

Although studies have been carried out to research autophagy in periodontitis, most of them only focused on one pathway or one molecule, and systematic research on autophagy on periodontitis through a large scale of samples and how autophagy could influence the immune characteristic are yet to be resolved. Thus, in our study, we systematically evaluated the role of autophagy in the immune microenvironment of periodontitis. The expression status of autophagy genes can well distinguish healthy and periodontitis samples. Using the most significantly altered autophagy genes, we managed to establish an autophagy-related model which can well distinguish samples between healthy and periodontitis. Next, to explore the relationship of immune microenvironment and periodontitis, the immunocyte, immune reaction and HLA status in periodontitis were revealed, and significant correlations were found with autophagy. To further understand how autophagy might shape the immune microenvironment in periodontitis, we applied unsupervised clustering of autophagy genes in periodontitis which identified 2 distinct autophagy-mediated regulation patterns in periodontitis. The 2 subtypes demonstrated diverse immune and clinical characteristics. Furthermore, WGCNA was applied to the gene expression values of the periodontitis samples and the brown module was found to be significantly correlated with subtype-2. Our findings indicate that autophagy plays a fundamental role in the microenvironment of periodontitis.

## RESULTS

### The landscape of autophagy gene alterations in periodontitis

**Figure 1:**
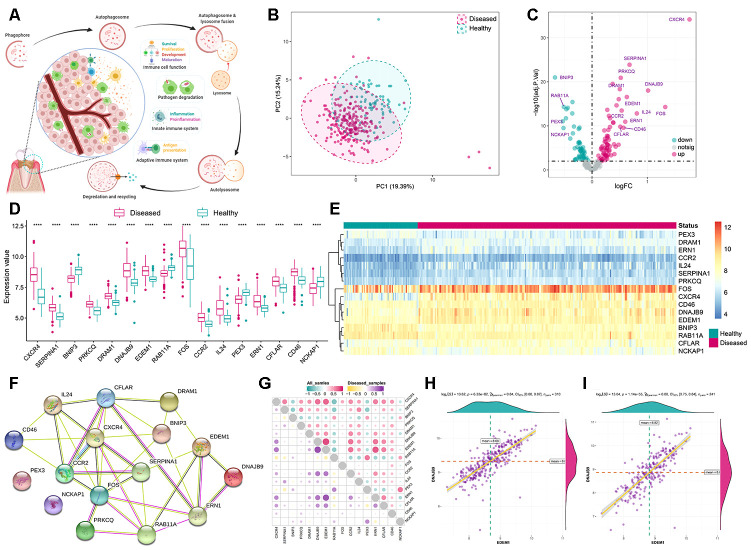
**Expression landscape autophagy genes in periodontitis.** (**A**) The overview of autophagy in regulating dynamic homeostasis of the immune microenvironment in periodontitis. (**B**) Principal component analysis (PCA) of 208 autophagy genes between healthy and periodontitis. The two first principal components (PC1, PC2) which could explain most of the variables are plotted, suggesting there are diversity regulation patterns of autophagy between healthy and periodontitis. (**C**) The volcano-plot shows the summary of expression changes of 208 autophagy genes between healthy and periodontitis samples and the most significant 16 autophagy genes are labeled. (**D**, **E**) The box-plot and heatmap-plot demonstrated the transcriptome expression status of 16 significantly dysregulated autophagy genes between healthy and periodontitis samples. (**F**) The 16 significant dysregulated autophagy gene protein-protein interactions are presented. (**G**) Correlations among the expression of 16 significantly dysregulated autophagy genes in all samples and periodontitis samples. (**H**, **I**) The two scatter-plots demonstrated the most correlated two autophagy genes: EDEM1 and DNAJB9.

We obtained 208 autophagy genes from http://www.autophagy.lu/ to explore their expression status in periodontitis. The underlying function of autophagy function in periodontitis immune microenvironment was summarized in Figure 1A. Principle component analysis (PCA) revealed a diverse expression patterns between healthy and periodontitis samples (Figure 1B). The differential analysis revealed that there were 16 significantly dysregulated autophagy genes whose expression alterations were presented in the boxplot and heatmap (Figure 1C–1E, Supplementary Table 1). To reveal the interactions between these autophagy genes, the protein-protein network was constructed (Figure 1F) and through correlation analysis, we found that EDEM1 and DNAJB9 were the most correlated autophagy genes (Figure 1G–1I).

### Autophagy genes can well distinguish healthy and periodontitis samples

**Figure 2:**
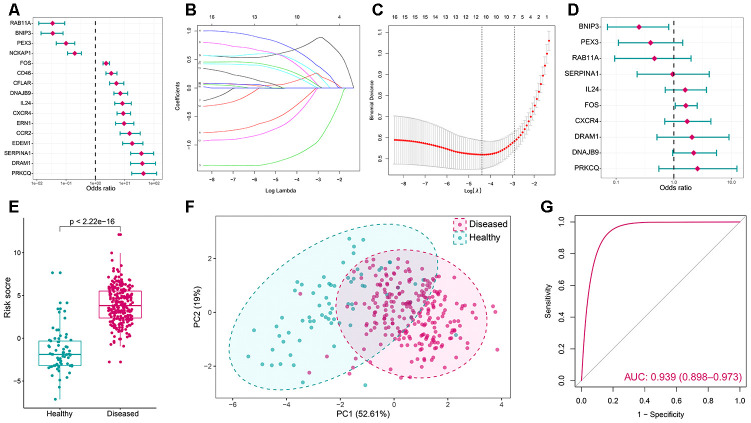
**Autophagy genes can distinguish healthy and periodontitis samples.** (**A**) Univariate logistic regression investigated the relationship between dysregulated autophagy genes and periodontitis. (**B**) Least absolute shrinkage and selection operator (LASSO) coefficient profiles of 16 periodontitis-related autophagy genes. (**C**) Ten-fold cross-validation for tuning parameter selection in the LASSO regression. The partial likelihood deviance is plotted against log (λ), where λ is the tuning parameter. Partial likelihood deviance values are shown, with error bars representing SE. The dotted vertical lines are drawn at the optimal values by minimum criteria and 1-SE criteria. (**D**) Distinguishing signature with 10 autophagy genes was developed by multivariate logistic regression and the risk scores for periodontitis were calculated. (**E**) The risk distribution between healthy and periodontitis, where periodontitis has a much higher risk score than healthy samples. (**F**) Principal component analysis (PCA) of 10 periodontitis-related autophagy genes between healthy and periodontitis. The two first principal components (PC1, PC2) which could explain most of the variables are plotted. (**G**) The discrimination ability for healthy and periodontitis samples by autophagy genes was analyzed by the ROC curve and evaluated by AUC value.

To find out crucial autophagy genes in periodontitis, a series of bioinformatic algorithms were employed on the 16 significantly dysregulated autophagy genes. To investigate the relationships between significantly altered autophagy genes and periodontitis, univariate logistic regression analysis was employed (Figure 2A, Supplementary Table 2). Next, LASSO regression was performed for feature selection and dimension reduction on the 16 significantly dysregulated autophagy genes and 10 periodontitis-related autophagy genes were found (Figure 2B, 2C). The 10 periodontitis-related autophagy genes were then passed onto multivariate logistic regression analysis for the model construction, and we obtained the risk scores for each of the samples (Figure 2D, 2E, Supplementary Table 3). Furthermore, PCA analysis demonstrated that the expression values of the 10 critical autophagy genes can well distinguish periodontitis and healthy samples (Figure 2F), suggesting autophagy gene FOS may make the greatest contribution to distinguish periodontitis samples from healthy (Supplementary Figure 1 and Supplementary Table 12). The receiver operating characteristic curve was plotted and the results showed that the autophagy model has excellent discrimination ability (Figure 2G). The validation was performed in another dataset GSE10334 which generated a similar result, which indicated the robustness of the model (Supplementary Figure 2).

### The correlation between periodontitis immune microenvironment and autophagy

**Figure 3:**
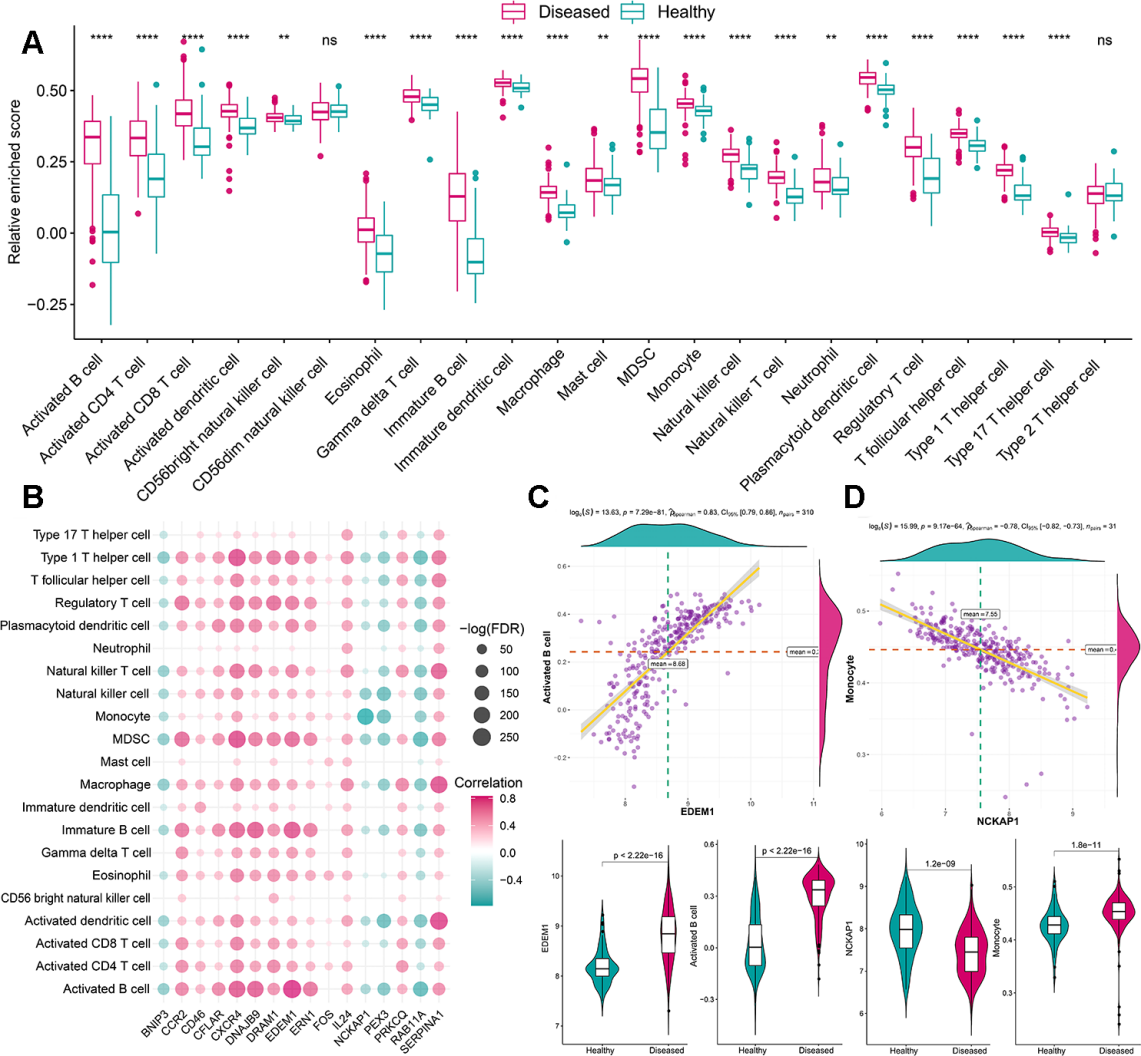
**The correlation between infiltrating immunocytes and autophagy genes.** (**A**) The difference in the abundance of each immune microenvironment infiltrating cells between healthy and periodontitis samples. (**B**) The dot-plot demonstrated the correlations between each dysregulated immune microenvironment infiltration cell type and each dysregulated autophagy genes. (**C**) The most positive correlated immunocyte-autophagy gene pairs are EDEM1-Activated B cell and the expression status or fraction status are presented by violin-plot at the left panel, indicating a higher expression of EDEM1 and a higher fraction of Activated B cell were found in periodontitis. (**D**) The most negatively correlated immunocyte-autophagy gene pairs are NCKAP1-Monocyte and the expression status or fraction status are presented by violin-plot at the right panel, indicating there is a lower expression of NCKAP1 in periodontitis and a higher level of the monocyte population.

**Figure 4:**
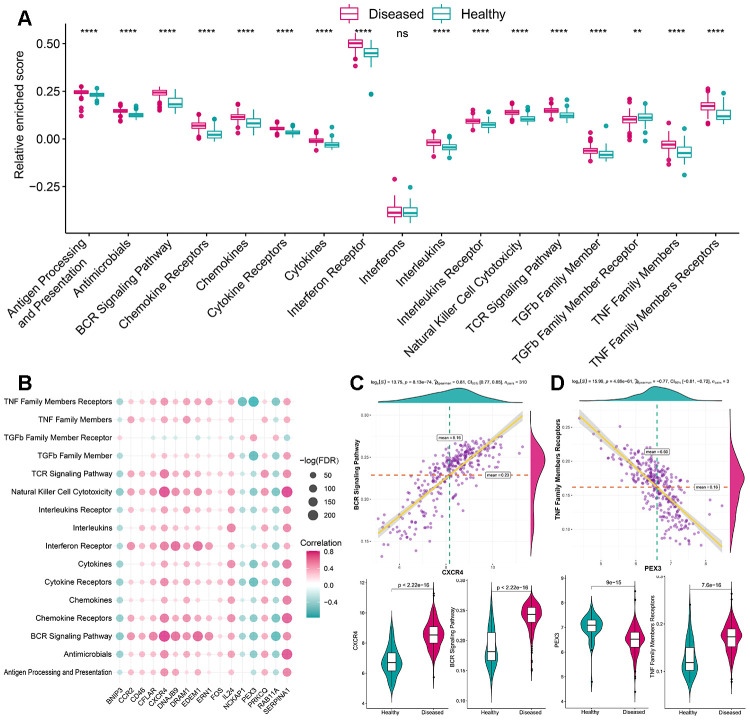
**The correlation between immune reaction gene-sets and autophagy genes.** (**A**) The difference in the activity of each immune reaction gene-set between healthy and periodontitis samples. (**B**) The dot-plot demonstrated the correlations between each dysregulated immune reaction gene-set and each dysregulated autophagy gene. (**C**) The most positive correlated pair is CXCR4-BCR Signaling Pathway and the expression status or activity status is presented by violin-plot at the left panel, indicating a higher expression of CXCR4 and more active BCR Signaling Pathway reaction were found in periodontitis. (**D**) The most negatively correlated pair is PEX3-TNF Family Members Receptors and the expression status or activity status is presented by violin-plot at the right panel, indicating a lower expression of PEX3 and more active TNF Family Members Receptors reactions in periodontitis.

**Figure 5:**
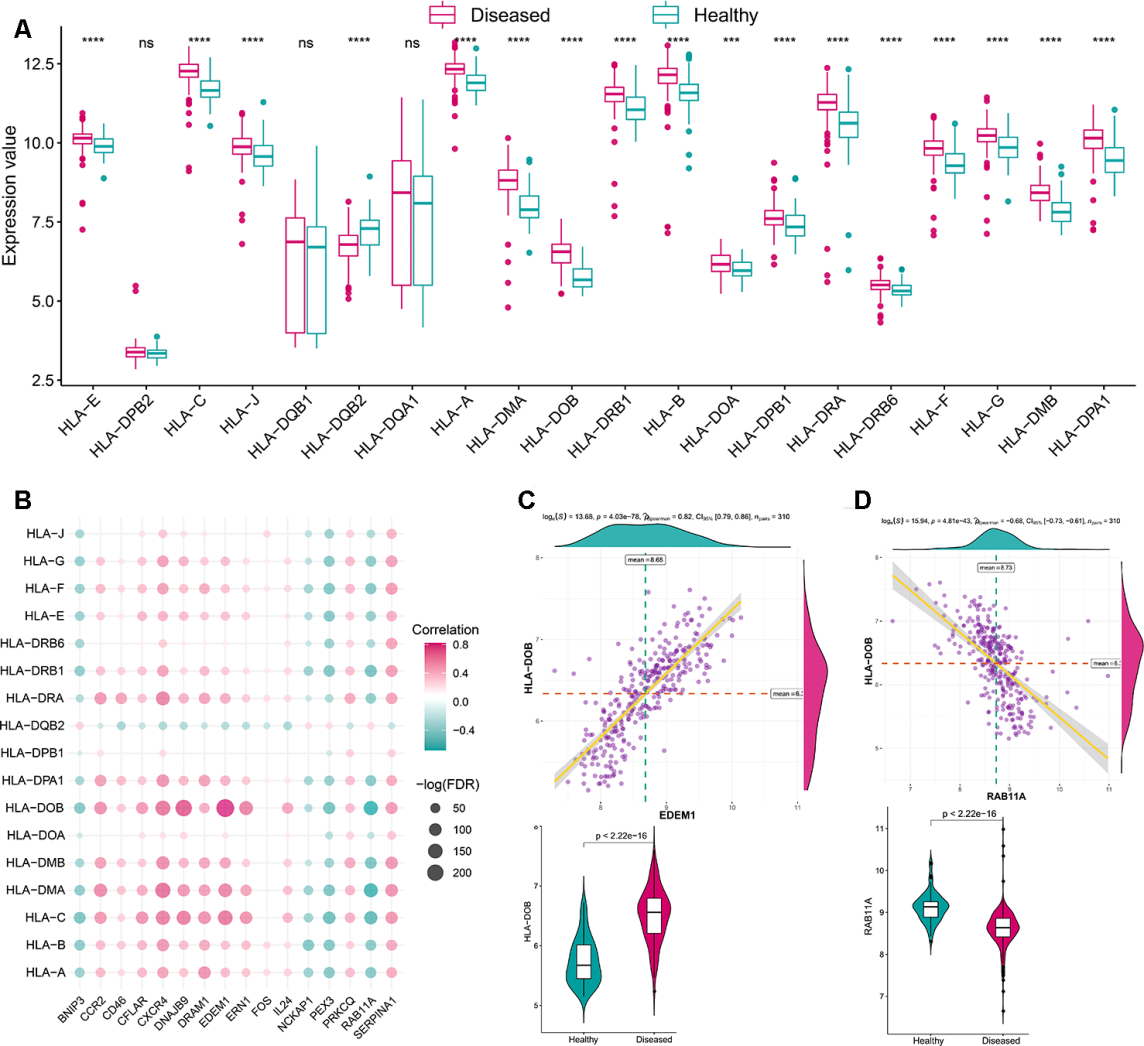
**The correlation between HLA and autophagy genes.** (**A**) The difference in the transcriptome expression of each HLA gene between healthy and periodontitis samples. (**B**) The dot-plot demonstrated the correlations between each dysregulated HLA gene and each dysregulated autophagy gene. (**C**) The most positive correlated HLA-autophagy pair is EDEM1-HLA DOB and the expression is presented by violin-plot at the left panel, indicating there is a higher expression of EDEM1 and HLA-DOB in periodontitis. (**D**) The most negatively correlated HLA-autophagy pair is RAB11A-HLADOB and the expression status is presented by violin-plot at the right panel, indicating low expression of RAB11A and higher expression of HLA-DOB in periodontitis.

As mentioned earlier, the immune microenvironment was crucial in the pathogenesis of periodontitis and was also closely linked to autophagy. Thus, to further explore their relationship, the overview of the immune microenvironment in periodontitis was portrayed. ssGSEA was applied to calculate the relative enrichment of each immunocyte in each sample, as was shown in Figure 3A, dramatic changes of immunocytes occurred in periodontitis samples, most of the immunocytes were upregulated in periodontitis such as activated B cell, suggesting a great change of immune microenvironment was happening in periodontitis. Using this immunocyte composition matrix we obtained, we can then explore the correlation of immunocyte with autophagy genes (Figure 3B, Supplementary Tables 4, 5). The most positively correlated immunocyte-autophagy gene pair is EDEM1-Activated B cell, and a higher expression of EDEM1 and a higher score of Activated B cell were found in periodontitis (Figure 3C); while the most negatively correlated pair is NCKAP1-Monocyte, and a lower expression of NCKAP1 and a higher level of Monocyte population could be found in periodontitis (Figure 3D). Likewise, the activity of immune-related pathways and expression levels of HLA was calculated and significant changes could be observed between healthy and periodontitis samples (Figures 4A, 5A). Their correlations with autophagy were also fully explored (Figures 4B, 5B, Supplementary Tables 6–9). For immune pathways, the most positively correlated pair is CXCR4-BCR Signaling Pathway, and a higher expression of CXCR4 and more BCR Signaling Pathway reaction were found in periodontitis (Figure 4C); while the most negatively correlated pair is PEX3-TNF Family Members Receptors and a lower expression of PEX3 and more active TNF Family Members Receptors reaction were found in periodontitis (Figure 4D). For HLA, the most positively correlated HLA-autophagy pair is EDEM1-HLADOB, and a higher expression of EDEM1 and HLA-DOB was observed in periodontitis (Figure 5C); while the most negatively correlated pair is RAB11A-HLADOB, indicating a lower expression of RAB11A and a higher expression of HLA-DOB could be observed in periodontitis (Figure 5D).

### Identification of different autophagy expression patterns

**Figure 6:**
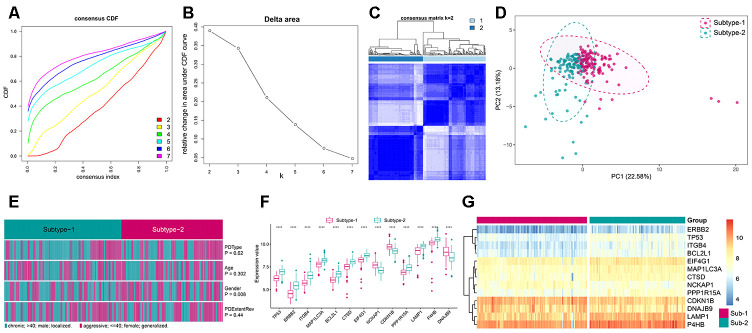
**Unsupervised clustering of 208 autophagy genes identifying 2 distinct autophagy-mediated regulation pattern subtypes in periodontitis.** (**A**) Consensus clustering cumulative distribution function (CDF) for k = 2–7. (**B**) Relative change in area under the CDF curve for k = 2–7. (**C**) Heatmap of the matrix of co-occurrence proportions for periodontitis samples. (**D**) Principal component analysis for the transcriptome profiles of 2 autophagy regulation patterns, showing a remarkable difference in transcriptome between different regulation patterns. (**E**) Comparing of age, gender, periodontitis range and periodontitis type among 2 autophagy regulation patterns. The heatmap illustrates the association of different clinical characters with the 2 subtypes. (**F**) The expression status of subtype-specific autophagy genes in the two subtypes. (**G**) Unsupervised clustering of 13 subtype-specific autophagy genes in the 2 regulation patterns.

To further explore the impact of autophagy in the immune microenvironment of periodontitis, we applied unsupervised clustering of 208 autophagy genes in periodontitis, which identified two subtypes with distinct autophagy expression patterns based on “majority rule”(Figure 6A–6C, Supplementary Figure 3, Supplementary Table 10). PCA analysis of the transcriptome profile of the two subtypes revealed that there was a remarkable difference in transcriptome between the two expression patterns (Figure 6D). Then, we compared the clinical characteristics between the two subtypes and found that gender significantly varied between the two subtypes, and there were more female in subtype-2 (Figure 6E). Next, we compared the expression of the subtype-specific autophagy genes in the two subtypes, and all of them significantly changed between the two subtypes (Figure 6F), and unsupervised clustering of 13 subtype-specific autophagy genes in the two subtypes demonstrated distinct expression patterns (Figure 6G).

### Distinct immune characteristics were observed in the two autophagy expression patterns

**Figure 7:**
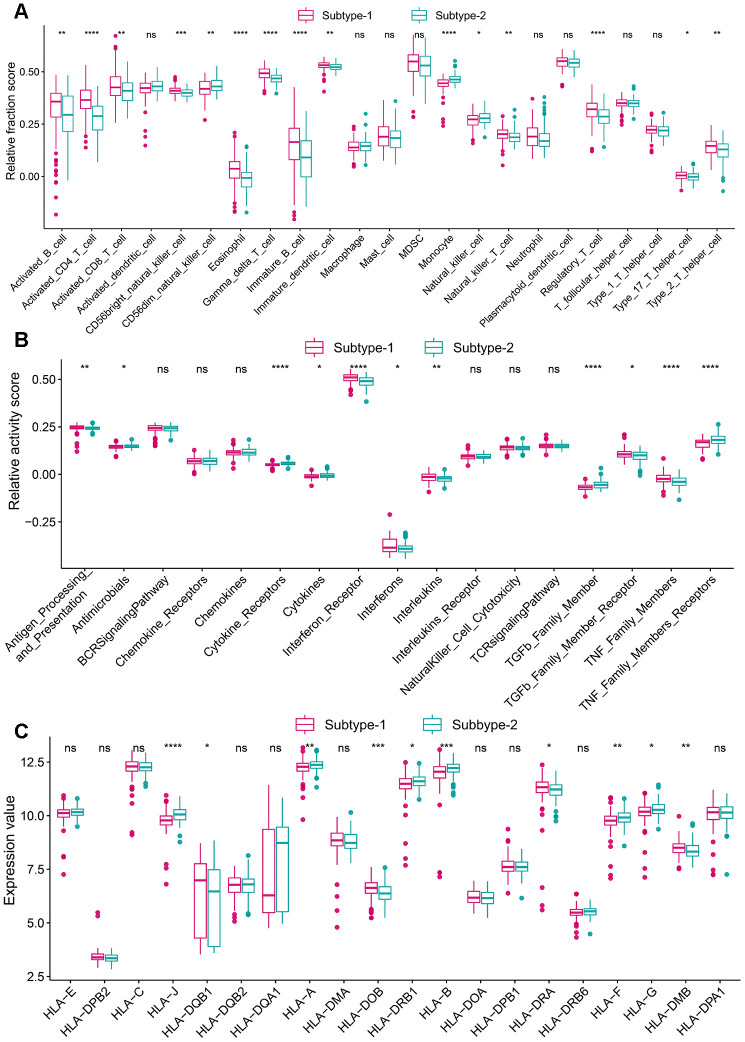
**Diversity of immune microenvironment characteristics among distinct autophagy-mediated regulation patterns.** (**A**) The abundance differences of each immune microenvironment infiltrating immunocytes in 2 autophagy regulation patterns. (**B**) The activity differences of each immune reaction gene-sets in 2 autophagy regulation patterns. (**C**) The expression differences of each HLA gene in 2 autophagy regulation patterns.

To explore the immune characteristics between the two subtypes, we compared the relative enrichment score of immunocytes, activity of immune pathways, and expression of HLA, and as expected, the two subtypes demonstrated very different immune characteristics, revealing autophagy-mediated distinct immune characteristics (Figure 7A–7C). For example, more activated CD4 T cells, and interferon receptors and lower expression of HLA-J were observed subtype-1. There are more infiltrating immunocytes, more active immune reactions and higher HLA gene expression, suggesting subtype-1 represents immune enrichment. Besides, immune characteristics among healthy and autophagy-based periodontitis subtypes were also investigated (Supplementary Figure 4). We also conducted a “gsva” algorithm to calculate the enrichment scores for autophagy subtypes and the relationship between the autophagy subtypes and periodontitis subtypes were investigated. The results revealed subtype-1 presented more macrophage, CMA, and mitophagy, while the subtype-2 is mainly related to microautophagy (Supplementary Figure 5).

### Biological functions behind autophagy expression patterns

**Figure 8:**
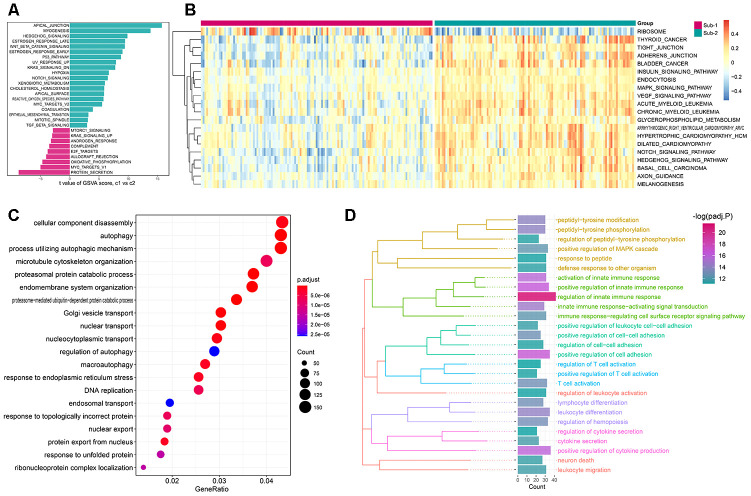
**The underlying biological characteristics diversity among 2 autophagy-mediated regulation patterns.** (**A**, **B**) The differences of the HALLMARKS pathway and KEGG pathway enrichment score between autophagy-mediated pattern 1 and pattern 2 (**A** for HALLMARKS pathway and B for KEGG pathway). (**C**) GO-BP functional enrichment analysis revealed the biological characteristics of autophagy phenotype-related genes. (**D**) The GO-BP enrichment analysis for the autophagy phenotype-related immune genes uncovers the relationship between autophagy and immune regulations. GO categories are grouped according to functions.

**Figure 9:**
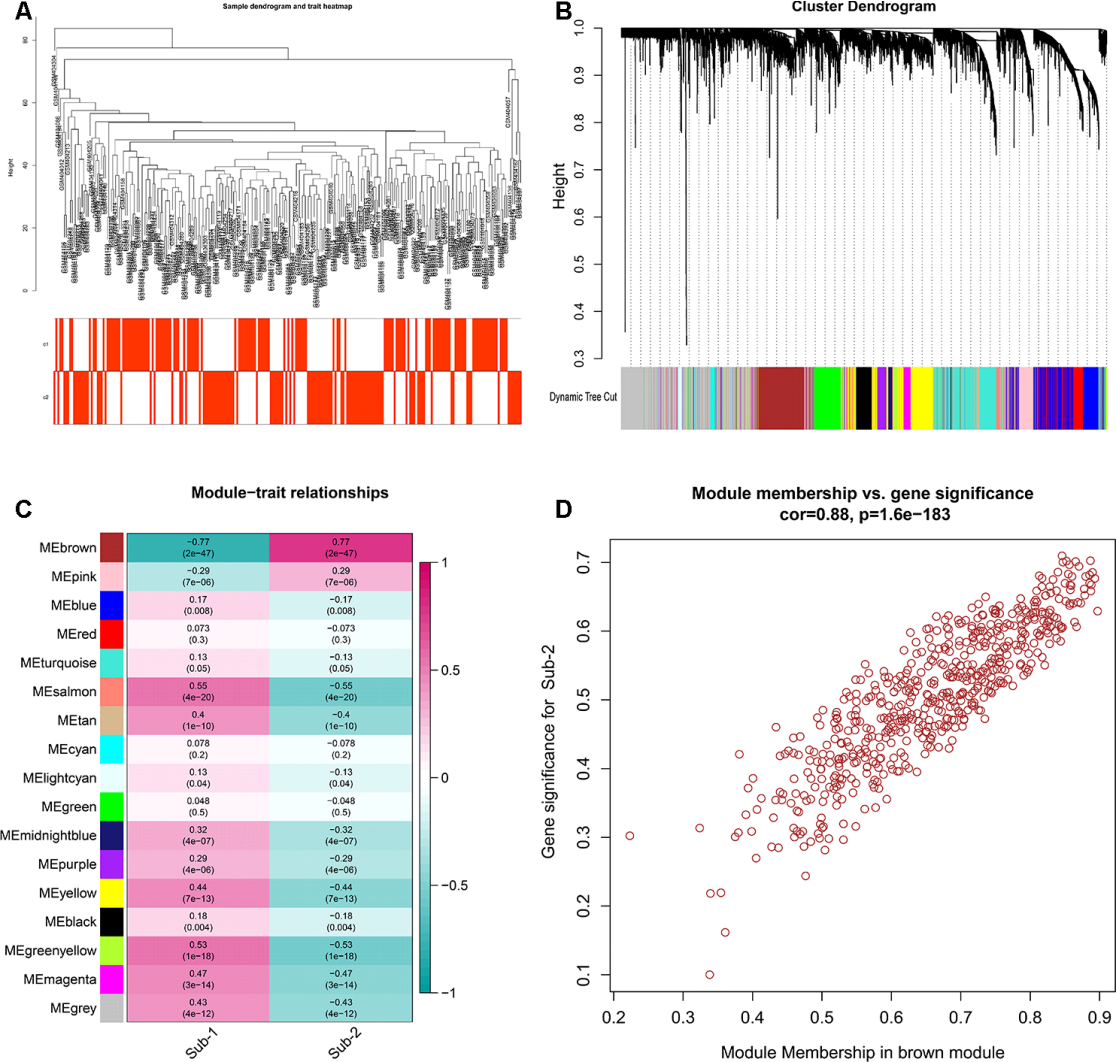
Gens and gene modules related to autophagy-mediated patterns (**A**) The sample clustering was based on the expression data of all samples. The top 25% of variation genes were used for the analysis by WGCNA and outlier samples were excluded. (**B**) Gene dendrogram obtained by average linkage hierarchical clustering. The color row underneath the dendrogram shows the module assignment determined by the Dynamic Tree Cut, in which 16 modules were identified. (**C**) Heatmap of the correlation between module eigengenes and the autophagy-mediated regulation patterns. (**D**) A scatterplot of gene significance (GS) for autophagy-mediated pattern-2 versus module membership (MM) in the brown module. GS and MM exhibit a very significant correlation, implying that hub genes of the green module also tend to be highly correlated with autophagy-mediated regulation pattern-2.

Apart from different immune characteristics in the two subtypes, we wonder if other distinct biological functions could be found in these two subtypes. GSVA analysis was employed to calculate the enrichment score of HALLMARK and KEGG pathway of the two subtypes, and a lot of pathways were enriched in subtype-2, such as APICAL JUNCTION and TIGHT JUNCTION (Figure 8A, 8B). The simplified GSEA GO-BP terms between the two subtypes with de-redundancy were also performed (Supplementary Figure 6, Supplementary Table 13). To further understand the role of autophagy in immunity, we identified autophagy phenotype-related genes which are the differentially expressed common genes between the two subtypes. 4309 genes were regarded as autophagy phenotype-related genes and GO-BP enrichment analysis revealed that they were mostly involved in cellular component disassembly, autophagy and process utilizing autophagic mechanism (Figure 8C, Supplementary Table 11). Next, the GO-BP enrichment analysis was carried out for the autophagy phenotype-related genes that participate in immunity, and regulation of innate immune response was significantly enriched, suggesting the fundamental role autophagy has in innate immune regulation (Figure 8D). Next, a comprehensive gene landscape correlated to each autophagy expression patterns were constructed, and gene-gene modules related to distinct autophagy regulations were identified by WGCNA (Figure 9A, 9B). 16 gene modules were determined and different expression pattern matched their related genes (Figure 9C), such as autophagy expression pattern-2 is closely related to genes in brown modules (Figure 9D). These results can shed light on the gene expression regulation network mediated by autophagy.

## DISCUSSION

Autophagy is a process in which cytoplasmic contents are being devoured and degraded by lysosome. It has long been recognized as a coping mechanism of eukaryotic cells under the stress of nutrition deprivation and immune response [13]. Being a bridge that links innate and adaptive immunity, autophagy is thought to play important roles in antigen presentation, maintenance of lymphocyte homeostasis, and regulation of cytokine production [14]. Dysregulation of innate and adaptive immune reactions plays fundamental roles in the etiology of periodontitis [15]. If the host’s immune response went out of control, it might generate an unceasing pathogenic cycle in which inflammatory response and dysbiosis reinforce each other, causing chronic inflammation and destruction in periodontium [16]. Knowing that autophagy is indispensable in immune reactions, we believe that autophagy must have a significant impact on shaping the immune microenvironment of periodontitis. Therefore, we aim to further dig into immunity in periodontitis from a new aspect of autophagy to see how it might shape the immune microenvironment of periodontitis. To answer these questions, we systematically investigated the autophagy expression patterns in the immune microenvironment of periodontitis. To reveal the effects of autophagy in immune microenvironment, including the composition of immunocytes and activity of immune related pathways, a series of bioinformatic algorithms were performed and we made the following discoveries. First, dysregulated autophagy genes were found in periodontitis which correlated and interacted with each other, revealing a regulatory network of autophagy in periodontitis. To see if dysregulated autophagy genes have well discrimination ability to distinguish periodontitis samples, univariate logistic regression analysis, LASSO regression analysis and multivariate regression analysis were performed to construct a classifier based on 10 periodontitis-related autophagy genes. Second, to catch a glimpse of the immune microenvironment of periodontitis, we used the ssGSEA algorithm to establish a matrix to evaluate the composition of immune cells, activity of immune pathways, and the expression of HLA genes were also taken into consideration. Then, their correlations with autophagy genes were fully explored. We found that the expression of CXCR4 was significantly positively correlated with Myeloid-derived suppressor cells (MDSC) and the expression of RAB11A were negatively correlated with activated dendritic cell. In colorectal cancer, it was found that overexpression of CXCR4 could promote the infiltration of myeloid-derived suppressor cells (MDSCs) [17]. MDSC functions as immune suppressor cells, whose population grows under the stimulus of chronic inflammation and in turn contributes to immunosuppression and oxidative stress [18]. Besides, we found that CXCR4 is positively correlated with BCR Signaling Pathway and PEX3 is negatively correlated with TNF Family Members Receptors; EDEM1 is positively correlated with HLADOB and RAB11A is negatively correlated with HLADOB. Some of these correlations haven’t been found in any other previous studies yet, which means that they might be novel approaches to understand the role of autophagy in periodontitis. Since a tight correlation was observed between autophagy and the immune microenvironment in periodontitis, we wonder if we cluster the samples according to their autophagy expression profile, the clusters might demonstrate distinct immune characteristics. The findings proved our point. The two subtypes were very different in terms of immunocyte composition, immune pathways and HLA gene expression. For instance, higher fractions of regulatory T cells were observed in subtype-1, and regulatory T cells were essential to ensure the immune homeostasis and control tissue damage during periodontitis, which could be the key to immunotherapy for the treatment of periodontitis [19]. The two autophagy subtypes harboring diverse immune profiles could support the hypothesis that autophagy was involved in the immune reactions in periodontitis. In detail, we conducted unsupervised clustering to separate the periodontitis samples based on the expression of autophagy genes, generating subtypes with the most distinct autophagy expression profiles. Furthermore, very distinct immune characteristics were also observed between the two subtypes with different autophagy expression patterns. It can be concluded that autophagy had a strong impact on the immune microenvironment so that different immune characteristics were demonstrated between the two subtypes. In addition, this classification strategy for the autophagy subtype can help us understand the underlying mechanism of immune regulation with autophagy so that precise therapeutic methods can be applied and periodontitis can be subtyped molecular level or immune level not only the phenotype level. Studies have shown that autophagy is active in Treg cells to support their lineage stability and survival fitness [20]. Significant changes of the TNF family and their receptors were observed between the two subtypes. The most famous member of the family is TNF-α, which is a pro-inflammatory cytokine that contribute to acute and chronic inflammation and tissue injury [21], it could promote osteoclast activity, thus enhance bone resorption in periodontitis [22]. In patients with rheumatoid arthritis, autophagy is activated by TNF-α, which induces osteoclast-mediated bone resorption [23]. To reveal the biological features that cause the difference between the 2 autophagy expression patterns, we employed the GSVA algorithm and GO-BP functional enrichment analysis on the two sub-types and autophagy phenotype related genes. Interestingly, we find the most significantly enriched pathway in the autophagy phenotype related immune genes was the regulation of innate immune response, which suggested the hypothesis that autophagy is the primary form of innate immunity for eukaryotic cells [10]. Besides, we also identified gene modules related to autophagy-mediated patterns.

Our study was the first to systematically analyze the relationship between autophagy and the immune microenvironment in periodontitis. And we have obtained findings that were either reported in other diseases or brand new and required attention. These findings could well enlighten the development of immunotherapy from the aspect of autophagy in periodontitis. Besides, we have identified two distinct autophagy expression patterns that are different from any other classification standards in periodontitis. The autophagy expression pattern could help us enhance the understanding of autophagy in periodontitis and how it could shape the immune microenvironment. We speculated that there are deep connections between autophagy and the immune microenvironment, and we believe that these findings could inspire researchers to further dig into autophagy in periodontitis and reveals the mysteries that are yet unsolved. However, we acknowledge that there are indeed some shortcomings in this study. First, our study mainly focused on methodology, an experiment in vitro and in vivo is needed to confirm these results. Second, the data of the periodontitis we have obtained was incomplete. Since follow-up information, precise clinical classification data and many other clinicopathological data were missing, relevant analysis cannot be performed so that we weren’t able to find out if there were other distinct clinicopathological characteristics between the 2 autophagy expression patterns. Last, the measurements for immunocytes and pathway activation are based on the GSVA score, which is calculated by gene expression at the mRNA level, and it cannot reflect the changes occurring on the protein level. This leads to poor performance in measurements of protein reaction-based pathway activation, for example, the inconsistency between the molecular pathway and directly related cellular activities (the enrichment of TCR Signaling Pathway was not significantly different between the two subtypes while significant differences in the enrichment scores of effector T cells were observed). Nevertheless, our findings indicated the strong impact autophagy has on the immune microenvironment of periodontitis, and have provided new insights into understanding the pathogenesis of periodontitis. Our study were the first to systematically reveal the underlying connections between autophagy and immune microenvironment in periodontitis, the findings might offer clues for other researchers to further uncover the mechanisms of autophagy in periodontitis.

## CONCLUSIONS

In conclusion, our study revealed the underlying mechanism of the impact of autophagy on the immune microenvironment of periodontitis. The systematic analysis of the diverse autophagy expression pattern will enhance the understanding of the pathogenesis of periodontitis and will inspire researchers to continue this direction of research.

## MATERIALS AND METHODS

### Data preparation

The data used in this study included 310 samples (69 healthy samples and 241 periodontitis samples), which came from 120 patients who underwent periodontal surgery in a previous study of Kebschull et al [24]. The sample processing and RNA extraction protocol were described in a previous study [24]. The gene expression was detected by Affymetrix Human Genome U133 Plus 2.0 Array microarray according to the manufacturer's instructions. The data was reserved in the GEO database under the serial number GSE16134 (https://www.ncbi.nlm.nih.gov/geo/query/acc.cgi?acc=gse16134). The data was obtained by the R package “GEOquery”. CEL files in the series were processed using a package named “RMA” in R using “justRMA” with default parameters. Probes were annotated as gene symbols, and probes without matching gene symbols or had multiple matching gene symbols were excluded. The gene expression of duplicate gene symbols was calculated as the median value. The expression value was processed by “normalizeBetweenArrays” in the R package “limma”. The 208 autophagy genes analyzed in this study were obtained from the database http://www.autophagy.lu/.

### Variation analysis of autophagy between periodontitis and healthy samples

The differential analysis of autophagy genes between healthy and periodontitis samples was carried out using the Wilcox test, and autophagy genes with adj.P.Val<0.01 and |logFC|>0.5 were considered as significantly dysregulated autophagy genes. The protein-protein network was constructed using the STRING database (https://string-db.org/). Correlation analysis of the 16 significantly dysregulated autophagy genes was carried out using Spearman correlation analysis in periodontitis samples as well as all samples. The periodontitis-related autophagy genes were identified by univariate logistic regression with the cut-off criteria of FDR < 0.01 and were then passed on to LASSO regression for feature selection and dimension reduction. Multivariate logistic regression was used to construct the periodontitis classifier using the periodontitis-related autophagy genes. Risk-scores are calculated by “predict” function according to gene expression and multivariate logistic coefficient, defined as the risk of suffering from periodontitis. ROC curve was plotted to assess the classification performance of the classifier.

### Correlation analysis between periodontitis immune microenvironment and autophagy

The relative enrichment of infiltrating immunocytes and the activity of immune pathways were determined by single-sample gene set enrichment analysis (ssGSEA). The gene sets used to determine the composition of immunocytes were obtained from the previous study [25]. The gene sets used to evaluate the activity of immune-related pathways were obtained from the database ImmPort (http://www.immport.org) [26]. The relative fraction of immunocytes, enrichment score of immune pathways and expression of HLA genes between healthy and periodontitis samples were compared using the Wilcox test. The correlation analysis of the relative fraction of immunocytes, enrichment score of immune pathways and expression of HLA genes between autophagy genes were done by Spearman correlation analysis.

### Identification of autophagy expression patterns

Unsupervised clustering analysis was conducted to identify distinct autophagy expression patterns according to the expression of 208 autophagy genes. A consensus clustering algorithm was used to evaluate the cluster numbers and robustness [27, 28]. The R package “ConsensuClusterPlus” implement the above steps for 1000 iterations for guaranteeing the robustness of classification [29]. The optimal number of clusters was evaluated by 30 indices according to the majority rule, which was performed by the “NbClust” function in the NbClust R package [30]. The comparison of expression of the periodontitis-related autophagy gene, immunocyte relative fraction, activity of immune pathways and expression of HLA genes between the two subtypes were conducted using the Wilcox test.

### Functional enrichment analysis of the two autophagy expression patterns

We used HALLMARKS and KEGG pathway to reflect biological changes that occurred in the two subtypes. Using the GSVA algorithm [31], the activity of each pathway was obtained, and was compared between two subtypes using the R package “limma”. Pathways with adjust adj.P.Val < 0.01 were considered to be significant. The gene-sets of “h.all.v7.0.symbols” and “c2.cp.kegg.v7.0.symbols” were downloaded from MSigDB database for GSVA analysis. The biological characteristics of autophagy phenotype-related genes and autophagy phenotype-related immune genes were uncovered by GO-BP enrichment analysis using the R package “clusterProfiler”.

### Identification of autophagy expression pattern related gene modules

To screen for autophagy expression pattern related genes, differentially expressed genes between two subtypes were identified using the R package “limma”. The criterion for DEGs was adjusted-p-value < 0.00001. The autophagy expression pattern related genes and gene modules were identified by WGCNA using the R package “WGCNA” [32, 33].

## Supplementary Material

Supplementary Figures

Supplementary Table 1

Supplementary Tables 2 and 3

Supplementary Table 4

Supplementary Tables 5 and 6

Supplementary Table 7

Supplementary Table 8

Supplementary Table 9

Supplementary Table 10

Supplementary Table 11

Supplementary Table 12

Supplementary Table 13
