# Supplementary material for: Autophagy-mediated regulation patterns contribute to the alterations of the immune microenvironment in periodontitis
Source: Aging (Albany NY). 2020 Dec 3;13(1):555–77. doi: 10.18632/aging.202165 (PMC7835039; doi:10.18632/aging.202165)
Supplement: Supplementary Figures [file aging-13-202165-s001.pdf]

SUPPLEMENTARY FIGURES

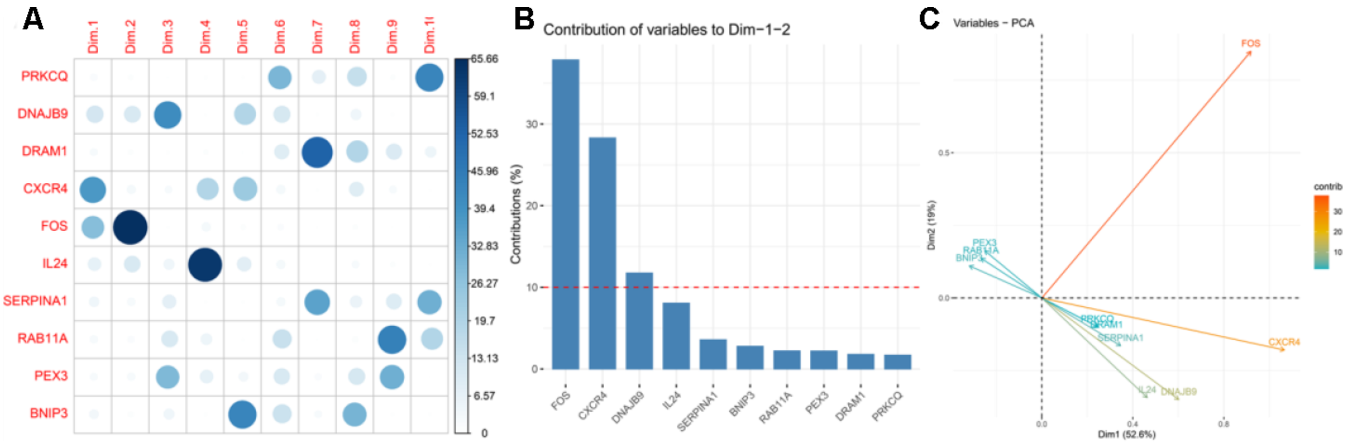

Supplementary Figure 1. Contribution of autophagy genes in PCA analysis. (**A**) The distribution dotplot for contribution of autophagy genes in every principle component. (**B**) The total contribution to PC1 and PC2 is calculated. (**C**) The most important (or, contributing) variables can be highlighted on the correlation plot.

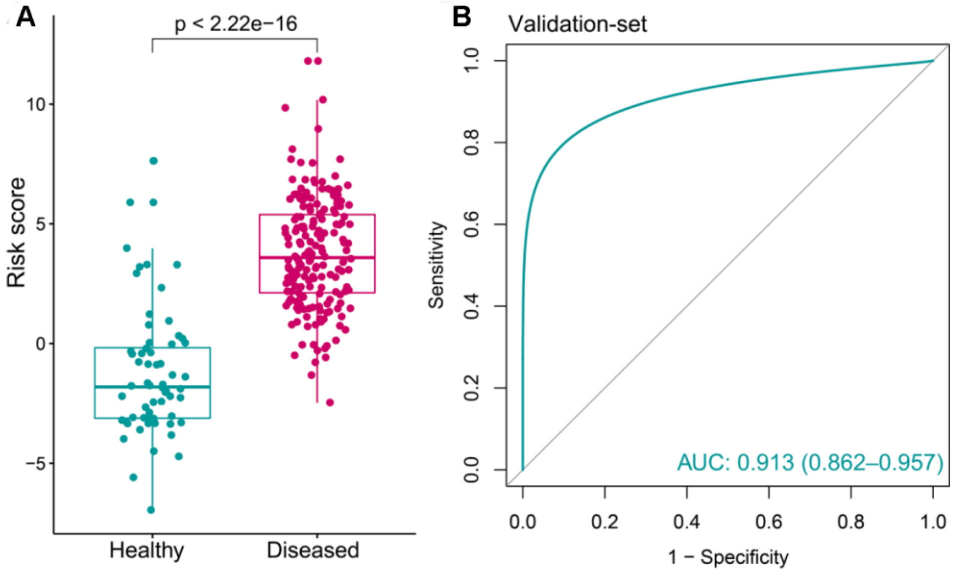

Supplementary Figure 2. External dataset validation by ten periodontitis-related autophagy genes generated from training set. (**A**) The risk distribution between healthy and periodontitis in validation set, in which the risk scores of periodontitis samples are much higher than that of healthy samples. (**B**) The discrimination ability of autophagy genes in validation set was analyzed by the ROC curve and evaluated by AUC value.

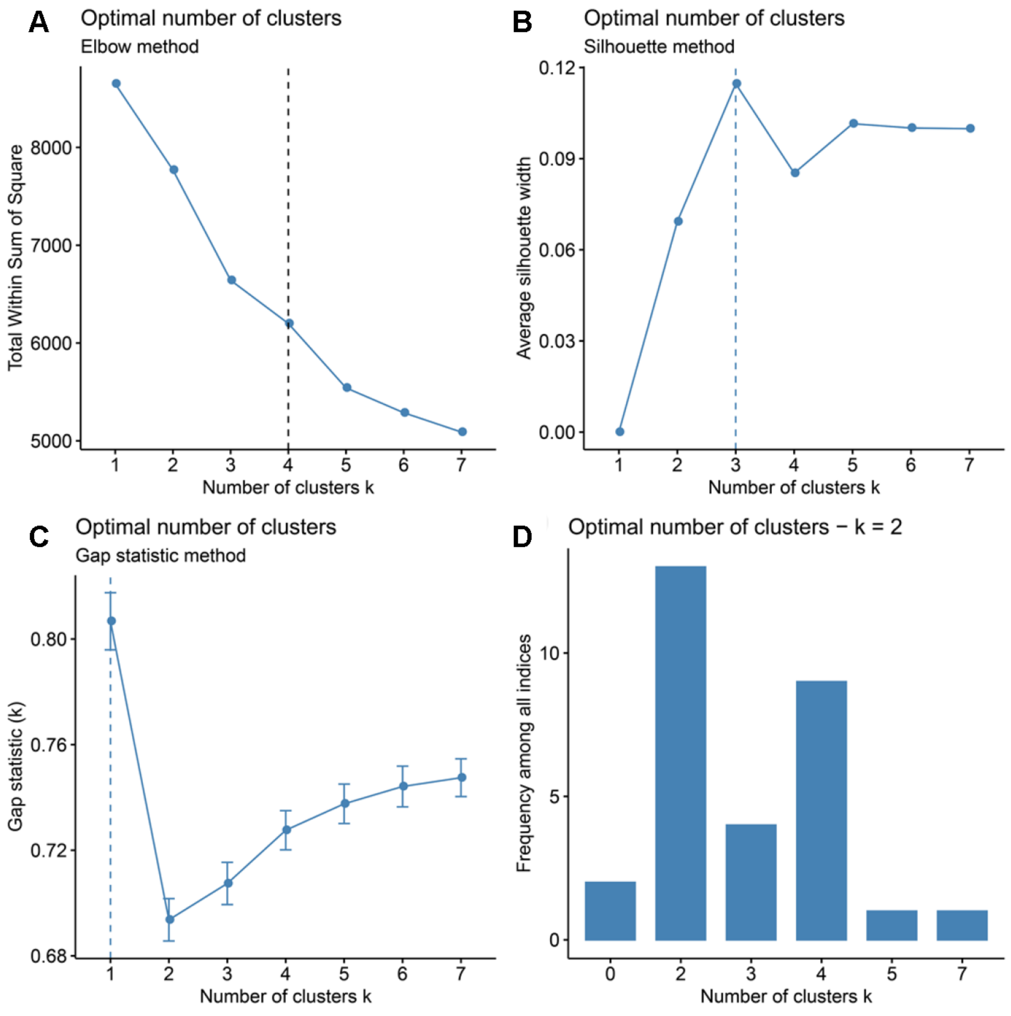

Supplementary Figure 3. (**A**) Elbow method suggesting 4 clusters solution may be the optimal. (**B**) Silhouette method suggesting 3 clusters solution may be the optimal. (**C**) Gap method suggesting 1 cluster solution may be the optimal. (**D**) Frequency distribution for 30 indices of choosing the best number of clusters. According to the majority rule, the best number of clusters is 2.

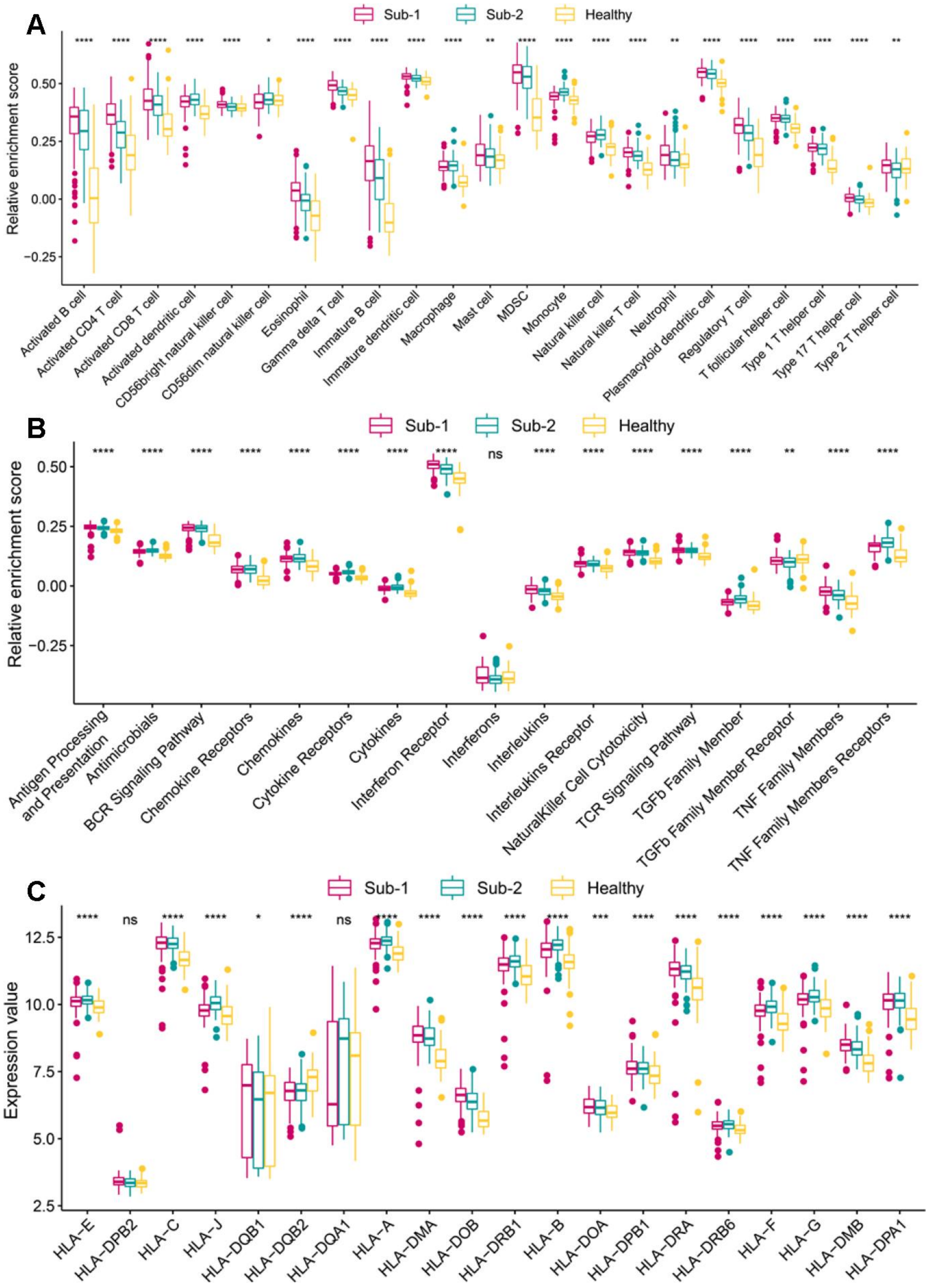

Supplementary Figure 4. The comparison of immune characteristics among healthy samples and autophagy-based periodontitis subtypes for infiltrating immunocytes abundance (**A**), immune reaction activity (**B**) and HLA gene expression (**C**).

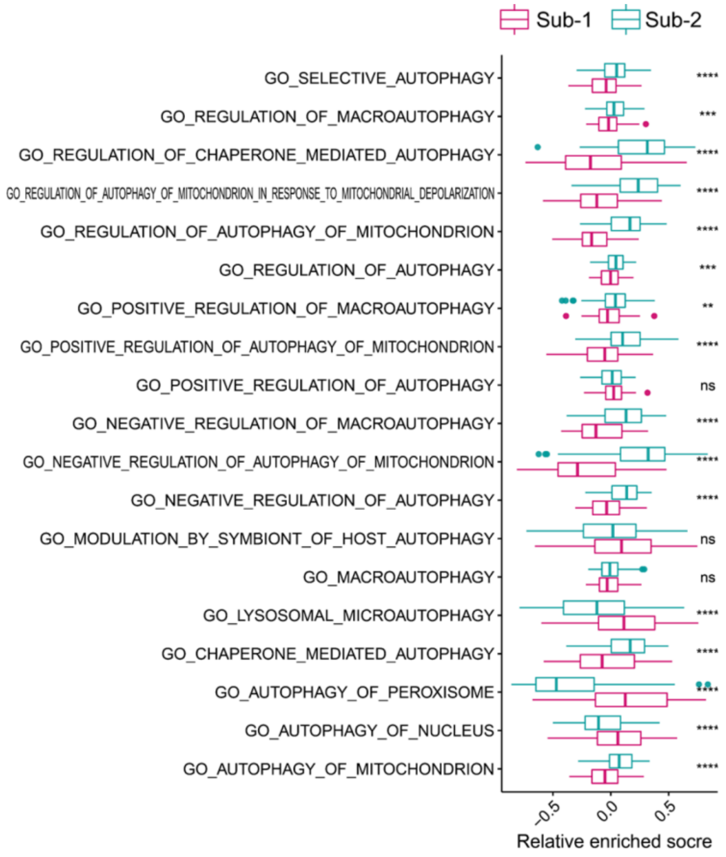

Supplementary Figure 5. The relationship between autophagy gene-based periodontitis subtypes and existing classifications of autophagy subtypes.

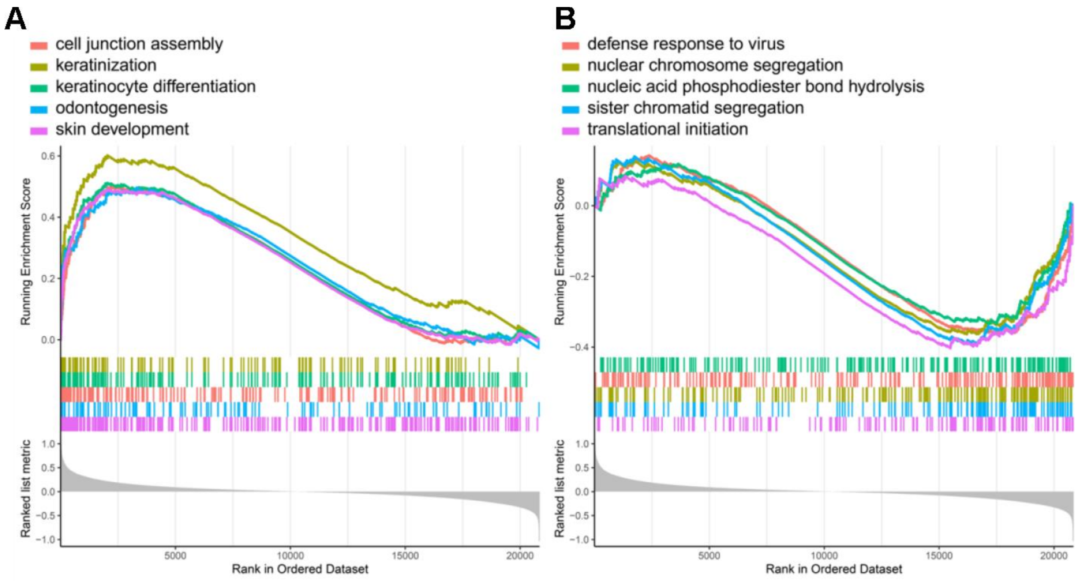

Supplementary Figure 6. GSEA revealed the difference of biological process between the two subtypes with de-redundancy for GO terms. (**A**) The top 5 significant GO terms for subtype-2. (**B**) The top 5 significant GO terms for subtype-1.
