# Supplementary material for: Autophagy-mediated regulation patterns contribute to the alterations of the immune microenvironment in periodontitis
Source: Aging (Albany NY). 2020 Dec 3;13(1):555–77. doi: 10.18632/aging.202165 (PMC7835039; doi:10.18632/aging.202165)
Supplement: Supplementary Tables 2 and 3 [file aging-13-202165-s003.pdf]

SUPPLEMENTARY TABLES

Supplementary Table 2. Univariate logistic regression results.

id	OR	low95	high95	pvalue	fdr
CXCR4	8.95905197	5.508081451	15.94238744	4.21E-16	6.73E-15
SERPINA1	37.1972771	16.01824489	97.39303017	3.08E-15	2.46E-14
BNIP3	0.034982189	0.0139458	0.078698436	2.53E-14	1.15E-13
PRKCQ	43.73270504	17.46588255	123.4575199	2.87E-14	1.15E-13
DRAM1	40.10742684	15.94380918	113.0215206	1.22E-13	3.91E-13
DNAJB9	7.128247166	4.305372072	12.67598588	8.17E-13	1.87E-12
EDEM1	18.06813529	8.580894317	42.00052636	7.36E-13	1.87E-12
RAB11A	0.034505725	0.011671209	0.0893268	8.25E-11	1.32E-10
FOS	2.32563562	1.831553852	3.027552655	3.80E-11	6.76E-11
CCR2	14.73484889	6.995711839	33.97905752	2.14E-11	4.29E-11
IL24	8.398271685	4.5837483	16.86532237	1.35E-10	1.96E-10
PEX3	0.099562649	0.046212042	0.197872389	4.53E-10	6.05E-10
ERN1	9.581845379	4.888393721	20.59546091	6.34E-10	7.81E-10
CFLAR	5.220795947	3.06131357	9.363578845	6.11E-09	6.52E-09
CD46	3.479525428	2.295185926	5.46201312	1.47E-08	1.47E-08
NCKAP1	0.197881267	0.112177806	0.333252775	4.75E-09	5.43E-09

Supplementary Table 3. Multivariate logistic regression results.

ID	OR	low95	high95	pvalue
CXCR4	1.703476996	0.695891318	4.405843902	0.255737151
SERPINA1	0.953392859	0.231013398	4.122617736	0.947745907
BNIP3	0.249283753	0.070428319	0.816072448	0.025651892
PRKCQ	2.575233452	0.5503442	12.47583488	0.232242842
DRAM1	2.065272898	0.510172942	9.058183167	0.319707889
DNAJB9	2.21023794	0.955796135	5.515358496	0.075930725
RAB11A	0.461663318	0.094176447	1.986802152	0.318746505
FOS	1.615067805	1.057860578	2.544333493	0.031683274
IL24	1.574290888	0.709086092	3.688380212	0.276347786
PEX3	0.395537241	0.109585244	1.428728845	0.154151451
