# Supplementary material for: Autophagy-mediated regulation patterns contribute to the alterations of the immune microenvironment in periodontitis
Source: Aging (Albany NY). 2020 Dec 3;13(1):555–77. doi: 10.18632/aging.202165 (PMC7835039; doi:10.18632/aging.202165)
Supplement: Supplementary Tables 5 and 6 [file aging-13-202165-s005.pdf]

SUPPLEMENTARY TABLES

Supplementary Table 5. Diversity of immunocytes between healthy and periodontitis samples.

ID	control	treat	FC	pvalue
Activated_B_cell	0.028682337	0.303307441	10.57471168	1.96E-24
Activated_CD4_T_cell	0.203188255	0.325499403	1.601959733	1.91E-15
Activated_CD8_T_cell	0.325510633	0.422979084	1.299432462	9.02E-17
Activated_dendritic_cell	0.373875382	0.425813289	1.138917699	3.38E-18
CD56bright_natural_killer_cell	0.396470546	0.40462237	1.020560983	0.001557049
Eosinophil	-0.07644482	0.010637701	-0.139155292	2.70E-13
Gamma_delta_T_cell	0.450834647	0.479424376	1.063415112	4.93E-09
Immature_B_cell	-0.073652561	0.118068177	-1.603042393	6.97E-21
Immature_dendritic_cell	0.509927853	0.526558794	1.032614302	6.67E-09
Macrophage	0.075804796	0.143624938	1.89466821	6.11E-24
Mast_cell	0.166162005	0.18754276	1.128674155	0.006638189
MDSC	0.369494303	0.52998994	1.434365659	6.91E-25
Monocyte	0.426397056	0.452043959	1.060147935	1.85E-11
Natural_killer_cell	0.219708722	0.273328792	1.244050709	4.00E-19
Natural_killer_T_cell	0.132520668	0.19501585	1.471588195	5.22E-22
Neutrophil	0.160179802	0.185920142	1.160696541	0.001640532
Plasmacytoid_dendritic_cell	0.502117698	0.543308302	1.082033763	8.03E-17
Regulatory_T_cell	0.1973633	0.299154638	1.515756162	3.78E-19
T_follicular_helper_cell	0.307432516	0.348413561	1.133300945	7.73E-19
Type_1_T_helper_cell	0.146449915	0.219138288	1.496336059	1.73E-22
Type_17_T_helper_cell	-0.016246414	0.001577097	-0.097073533	1.27E-08

Supplementary Table 6. Diversity of immune reaction gene-sets between healthy and periodontitis samples.

ID	control	treat	FC	pvalue
Antigen_Processing_and_Presentation	0.232829877	0.244056786	1.048219366	1.63E-11
Antimicrobials	0.126458956	0.146051936	1.154935491	2.33E-19
BCRSignalingPathway	0.189246934	0.239929328	1.26781091	3.77E-24
Chemokine_Receptors	0.026075673	0.069281699	2.656947727	4.14E-21
Chemokines	0.083089047	0.115883381	1.394689002	1.59E-15
Cytokine_Receptors	0.035330253	0.054365591	1.538782966	2.50E-20
Cytokines	-0.028409514	-0.007951884	0.279902151	3.46E-17
Interferon_Receptor	0.447356591	0.497679544	1.112489576	1.28E-19
Interleukins	-0.04306784	-0.019413374	0.450762661	4.14E-13
Interleukins_Receptor	0.074911976	0.093326464	1.245815001	7.93E-13
NaturalKiller_Cell_Cytotoxicity	0.107220427	0.139171684	1.29799598	1.15E-22
TCRsignalingPathway	0.12608018	0.149229687	1.183609404	1.48E-17
TGFb_Family_Member	-0.079328228	-0.060743855	0.765728118	2.42E-10
TGFb_Family_Member_Receptor	0.110681235	0.101014205	0.912658812	0.005018149
TNF_Family_Members	-0.067819813	-0.030722486	0.453001626	4.61E-12
TNF_Family_Members_Receptors	0.129777052	0.171192354	1.31912654	7.56E-16
