# Supplementary material for: Autophagy-mediated regulation patterns contribute to the alterations of the immune microenvironment in periodontitis
Source: Aging (Albany NY). 2020 Dec 3;13(1):555–77. doi: 10.18632/aging.202165 (PMC7835039; doi:10.18632/aging.202165)
Supplement: Supplementary Table 8 [file aging-13-202165-s007.pdf]

SUPPLEMENTARY TABLE

Supplementary Table 8. Diversity of HLA gene expression between healthy and periodontitis samples.

ID	control	treat	FC	pvalue
HLA-E	9.900019815	10.11765558	1.021983367	2.58E-08
HLA-C	11.69971094	12.22933332	1.045267989	1.41E-18
HLA-J	9.613263411	9.882935586	1.028052095	1.00E-06
HLA-DQB2	7.20374812	6.728334111	0.934004632	7.14E-09
HLA-A	11.9206991	12.29871027	1.031710486	1.18E-13
HLA-DMA	7.984399658	8.792908871	1.101261115	2.13E-21
HLA-DOB	5.742159729	6.504613522	1.132781711	4.02E-23
HLA-DRB1	11.09291326	11.50098747	1.036786929	2.46E-12
HLA-B	11.56687536	12.04355889	1.041211088	1.12E-12
HLA-DOA	5.999200736	6.189431203	1.031709302	0.000198157
HLA-DPB1	7.367720578	7.646295694	1.037810217	1.83E-07
HLA-DRA	10.54226396	11.19533093	1.061947507	1.09E-14
HLA-DRB6	5.370241867	5.509055221	1.025848622	2.16E-06
HLA-F	9.349617249	9.805435584	1.04875262	2.67E-13
HLA-G	9.848227181	10.22515294	1.038273463	3.07E-11
HLA-DMB	7.885631024	8.430186322	1.069056655	1.55E-15
HLA-DPA1	9.46803567	10.07641674	1.064256313	9.78E-15
