# Supplementary material for: Autophagy-mediated regulation patterns contribute to the alterations of the immune microenvironment in periodontitis
Source: Aging (Albany NY). 2020 Dec 3;13(1):555–77. doi: 10.18632/aging.202165 (PMC7835039; doi:10.18632/aging.202165)
Supplement: Supplementary Table 12 [file aging-13-202165-s011.pdf]

SUPPLEMENTARY TABLE

Supplementary Table 12. The full list of principle component in PCA analysis of Figure 1B.

	PC1	PC2	PC3	PC4	PC5	PC6	PC7	PC8	PC9	PC10
PRKCQ	0.1397	-0.09422	0.022391	-0.06532	0.048928	-0.54474	0.273899	-0.39277	-0.08853	0.656177
DNAJB9	0.345465	-0.33409	-0.63874	-0.04296	0.440131	0.340012	0.063081	-0.21405	0.001548	-0.0088
DRAM1	0.14386	-0.09659	0.039521	-0.08886	-0.02423	-0.29794	-0.72556	-0.44278	0.31688	-0.21966
CXCR4	0.611874	-0.17105	-0.1426	0.44258	-0.48675	-0.17527	-0.0626	0.293805	-0.14555	-0.03275
FOS	0.527227	0.81033	0.038151	-0.17636	0.103776	0.115244	0.025634	-0.08867	0.012183	0.011059
IL24	0.265895	-0.32752	0.213464	-0.79559	-0.28834	0.171744	0.003583	0.141461	-0.09415	0.044437
SERPINA1	0.198778	-0.15699	0.276432	0.042715	0.047002	-0.17034	0.593059	-0.24131	0.312026	-0.56486
RAB11A	-0.15361	0.130599	-0.33015	-0.2238	-0.05045	-0.38302	0.032281	-0.11086	-0.66425	-0.44322
PEX3	-0.14421	0.155415	-0.54113	-0.26589	-0.18659	-0.32471	0.132196	0.335942	0.566407	0.011688
BNIP3	-0.18297	0.105645	-0.21059	0.058722	-0.65795	0.379767	0.140478	-0.55343	0.046242	0.047822
